# EHR4CR central workbench

**DOI:** 10.1186/2043-9113-5-S1-S13

**Published:** 2015-05-22

**Authors:** David Voets

**Affiliations:** 1 Custodix, Kortrijkse steenweg 214, bus 3, 9830 Sint-Martens-Latem, Belgium

## Characterisation

Tool, protocol feasibility, recruitment process, electronic data capture, multi-centric clinical trials.

## Description

**Figure 1:**
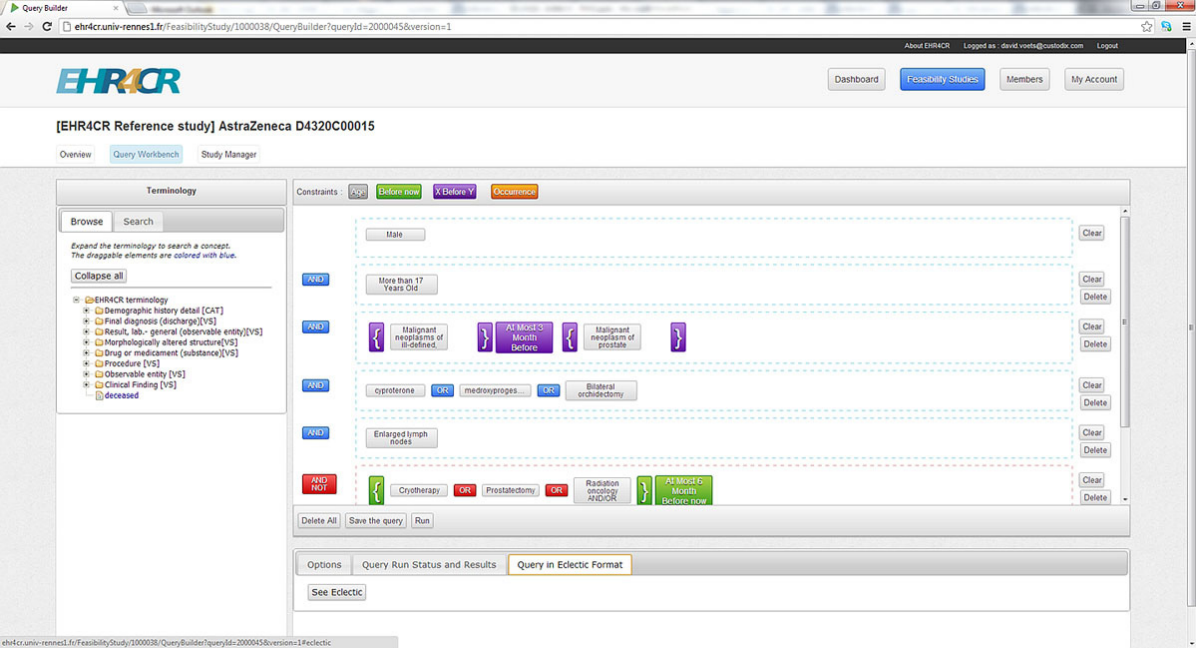
User interface of the central workbench showing different eligibility criteria connected by AND (blue fields).

The EHR4CR central query workbench supports pharma and CRO users in various stages of the multi-centric clinical trial lifecycle ranging from protocol feasibility, over subject identification and recruitment to clinical trial execution and adverse event reporting. It offers an intuitive graphical user interface for building, managing and executing formalized eligibility criteria queries (Figure 1) to support protocol feasibility studies using real-life data residing in distributed clinical data warehouses as evidence. As these data warehouses reside at the clinical sites and no patient-level information is disclosed (only patient counts aggregated over demographic categories), patient privacy is respected.

The EHR4CR central workbench facilitates the patient recruitment process by offering workflow-driven administration and monitoring of the recruitment process for multi-centric trials. The study manager can select clinical sites of interest and invite them to participate in the study. Study metadata including protocol definition and formalized eligibility criteria queries are exchanged and synchronized with the participating clinical sites. The overall study status and individual clinical site participation status are continuously monitored and updated. The study manager is automatically informed about relevant changes occurring at each of the engaged clinical sites such as changes in the number of potential candidate patients, the number of consenting patients, patients in screening, included or excluded patients at each site.

In order to support clinical trial execution workflows, the EHR4CR central workbench will provide “Retrieve Form for Data Capture” (IHE-RFD) [1] capabilities (Form Manager, Form Receiver) to allow EDC systems to retrieve annotated eCRF forms that allow auto-population directly from the local EHR or clinical data warehouse.

## Status of development

Protocol feasibility functionality has been evaluated in three separate UAT rounds involving specialists from pharma and 11 pilot clinical sites. Subject identification and recruitment functionality is being evaluated. Support for clinical trial execution is still in development (November 2014).

## Users

Pharma, CRO (study designers, study managers).

## Links

http://www.ehr4cr.eu
